# Postsynaptic p47phox regulates long-term depression in the hippocampus

**DOI:** 10.1038/s41421-018-0046-x

**Published:** 2018-08-28

**Authors:** Jee Hyun Yi, Dong Hyun Kim, Thomas M. Piers, Seung Chan Kim, Daniel J. Whitcomb, Philip Regan, Kwangwook Cho

**Affiliations:** 1 Henry Wellcome Laboratories for Integrative Neuroscience and Endocrinology, Bristol Medical School, Faculty of Health Sciences, University of Bristol, Whitson Street, Bristol, BS1 3NY UK; 2 UK-Dementia Research Institute, Department of Basic and Clinical Neuroscience, Maurice Wohl Clinical Neuroscience Institute, King’s College London, London, SE5 9NU UK

## Abstract

It is well documented that reactive oxygen species (ROS) affects neurodegeneration in the brain. Several studies also implicate ROS in the regulation of synapse function and learning and memory processes, although the precise source of ROS generation within these contexts remains to be further explored. Here we show that postsynaptic superoxide generation through PKCζ-activated NADPH oxidase 2 (NOX2) is critical for long-term depression (LTD) of synaptic transmission in the CA1–Shaffer collateral synapse of the rat hippocampus. Specifically, PKCζ-dependent phosphorylation of p47phox at serine 316, a NOX2 regulatory subunit, is required for LTD but is not necessary for long-term potentiation (LTP). Our data suggest that postsynaptic p47phox phosphorylation at serine 316 is a key upstream determinant for LTD and synapse weakening.

## Introduction

Synapse weakening is part of a group of physiological processes referred to as synaptic plasticity, which govern changes in synaptic function in response to neuronal activity, and are thought to represent the cellular and molecular mechanisms of learning and memory^1^. On the other hand, aberrant activation of synapse weakening signalling pathways has been reported in several Alzheimer’s disease (AD) models^2–4^, suggesting that these signalling pathways represent a crucial interplay between physiology and the onset of disease-associated pathophysiology. Mounting evidence suggests that apoptotic signalling cascades, including caspase-3 and glycogen synthase kinase 3β (GSK-3β) activation, are centrally involved in physiological and pathophysiological forms of synapse weakening, manifest through postsynaptic α-amino-3-hydroxy-5-methyl-4-isoxazolepropionic acid receptor (AMPAR) endocytosis and long-term depression (LTD) of synaptic transmission^4–6^. However, how these signals are first initiated is unknown.

Reactive oxygen species (ROS) are not only well-known upstream regulators of neuronal apoptosis^7^ and neurodegenerative signals^8^ but are also known to play important regulatory roles in aspects of neuronal physiology, including synaptic plasticity and synapse weakening^9–13^. ROS can originate from numerous sources to affect synaptic plasticity, including presynaptic neurons, postsynaptic neurons and microglia^11, 13^. However, to date there has been no characterization or elucidation of the precise mechanisms of ROS generation that regulate synaptic plasticity.

Here, we examined the production of postsynaptic ROS during LTD, revealing a key role for postsynaptic ROS production via NADPH oxidase 2 (NOX2). Crucially, we find that the activity of postsynaptic protein kinase C zeta (PKCζ) is also required for LTD and identify the phosphorylation of p47phox at serine 316 as a necessary step in this pathway. Therefore, our results uncover a role for a specific postsynaptic ROS production pathway in activity-dependent synapse weakening.

## Results

### Postsynaptic superoxide is required for LTD in the CA1 of the hippocampus

**Fig. 1:**
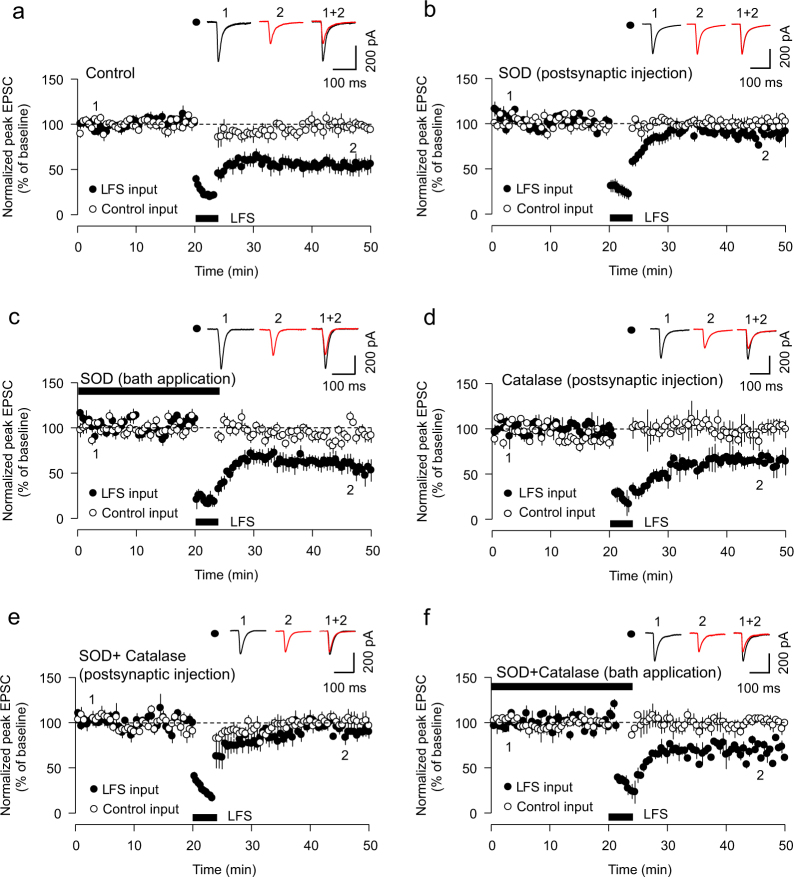
Postsynaptic superoxide is required for LTD expression. **a** Low frequency stimulation (LFS) induces LTD in patch clamp recording mode in CA1–Schaffer collateral synapses in rat hippocampal acute slices (*n* = 6). **b** Postsynaptic injection of SOD (300 units/ml) blocked LTD (*n* = 5). **c** Bath application of SOD has no effect on LTD expression (*n* = 5). **d** Postsynaptic infusion of catalase (300 units/ml, 20 min) has no effect on LTD (*n* = 5). **e** Postsynaptic infusion of SOD and catalase inhibits LTD (*n* = 6). **f** Bath perfusion of SOD and catalase has no effect on LTD (*n* = 6). Symbols and error bars indicate mean ± SEM

Superoxide ions are one of the primary forms of ROS and are known to be elevated in neurons following the activation of *N*-methyl-d-aspartate receptors (NMDARs)^14–16^. We therefore hypothesized that intra-neuronal superoxide radicals are upstream regulators of NMDAR-dependent forms of synaptic plasticity. To address this, we analysed the effects of superoxide dismutase (SOD), a class of endogenous enzymes that catalyse superoxide dismutation^17^, on an NMDAR-dependent form of LTD in rat hippocampal acute slices^18^. Accordingly, whilst application of low-frequency electric stimulation (LFS) during whole-cell patch clamp recording readily induced LTD in CA1–Schaffer collateral synapses (52.8 ± 9.0% of baseline, *p* = 0.002 vs. control input, Fig. 1a), postsynaptic infusion of SOD (300 units/ml) through the patch pipette blocked LTD (89.5 ± 8.7% of baseline, *p* = 0.115 vs. control input, Fig. 1b). To determine whether the superoxide radicals involved in this form of LTD could originate from an extracellular source, such as microglia^13^, we bath applied SOD. Given that SOD has poor membrane permeability^19^, extracellular bath application of the enzyme will catalyse extracellular superoxide dismutation without affecting intracellularly generated superoxide. Extracellular SOD application had no effect on LTD (57.5 ± 9.9% of baseline, *p* = 0.004 vs. control input, Fig. 1c), suggesting that postsynaptic intracellular superoxide, specifically, is critical for LTD expression. Since hydrogen peroxide (H_2_O_2_), another ROS, can be a product of SOD catalysis of superoxide dismutation and is also implicated in synaptic plasticity^20^, we tested whether H_2_O_2_ is required for LTD. Postsynaptic infusion of catalase (300 units/ml), an enzyme that catalyses the decomposition of H_2_O_2_ to water and oxygen, had no effect on LTD (66.8 ± 4.7% of baseline, *p* = 0.002 vs. control input, Fig. 1d). We also tested whether H_2_O_2_ may be inhibiting NMDAR-LTD, which could provide an alternative explanation for the inhibition of LTD induced by SOD injection. To test this, we postsynaptically injected SOD along with catalase, thereby scavenging the H_2_O_2_ product of the SOD reaction. In this experiment, LTD was also blocked (SOD+catalase, 90.1 ± 4.8% of baseline, *p* = 0.150 vs. baseline, Fig. 1e). In bath application experiments, SOD and catalase co-treatment did not affect LTD (SOD+catalase, 68.3 ± 1.2% of baseline, *p* < 0.001 vs. baseline, Fig. 1f). Together, these results suggest that postsynaptic intracellular superoxide is required for NMDAR-dependent hippocampal LTD and that H_2_O_2_ itself is neither required for nor an inhibitor of LTD.

### NOX2 regulates LTD

**Fig. 2:**
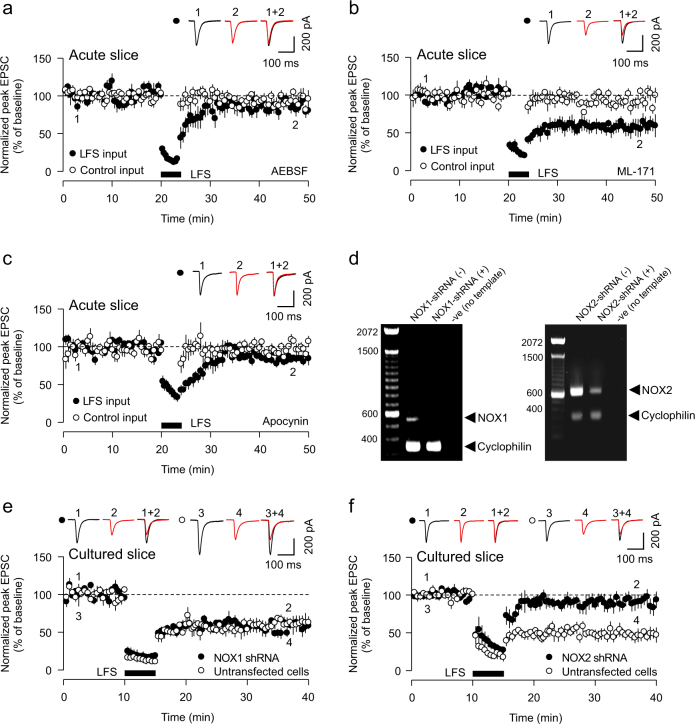
Neuronal NADPH oxidase 2 (NOX2) is required for LTD expression. **a** Postsynaptic infusion of the NADPH oxidase inhibitor AEBSF (20 μM) blocks LTD (*n* = 5) in CA1 neurons from acute hippocampal slices. **b** Postsynaptic infusion of the NOX1 inhibitor ML-171 (3 μM) has no effect on LTD (*n* = 5). **c** Postsynaptic infusion of the NOX2 inhibitor apocynin (100 μM) blocks LTD (*n* = 6). **d** Single-cell PCR assays demonstrate efficient knockdown of NOX1 (left) and NOX2 (right) with their respective shRNAs in CA1 neurons of organotypic hippocampal slices. **e** LTD is readily inducible in both NOX1 shRNA-transfected cells (filled circle, *n* = 5) and untransfected control cells (open circle, *n* = 6). **f** LTD is abolished in neurons biolistically transfected with NOX2 shRNA (filled circle, *n* = 5), whilst LTD is induced in untransfected cells (open circle, *n* = 5). All data points represent mean ± SEM. Representative EPSC single traces are provided in inset from the time points indicated

While several ROS-inducing mechanisms are present in neurons, emerging evidence suggests that superoxide production post-NMDAR activation is catalysed by NADPH oxidase (NOX)^15, 16^. NOX is a membrane-bound enzymatic complex responsible for the production of ROS and is present in neurons where it can localize to synapses^21^. Specifically, NOX2, commonly known as the prototypical NOX, has been suggested as the primary regulator of NMDAR-activated superoxide generation^15^. While NOX1–4 are reported to be expressed in the brain, NOX3 constitutively generates superoxide without stimulation^22^ and NOX4 constitutively produces H_2_O_2_^23^, an ROS that appears not to be involved in LTD. We therefore predicted that postsynaptic NOX1 and 2 are the most likely candidates for superoxide production during LTD. To test a requirement for these NOX isoforms for LTD, we utilized postsynaptic infusion of NOX inhibitors. Postsynaptic infusion of AEBSF (20 µM), a non-selective inhibitor of NOX^24^, blocked LTD expression in CA1 neurons of acute hippocampal slices (90.4 ± 6.0% of baseline, *p* = 0.146 vs. control input, Fig. 2a), whilst postsynaptic infusion of the specific NOX1 inhibitor ML-171 (3 μM)^25^ had no effect on LTD (58.9 ± 8.6% of baseline, *p* = 0.006 vs. control input, Fig. 2b). Postsynaptic infusion of apocynin (100 µM), a putative but non-selective inhibitor of NOX2^26^, significantly impaired LTD expression (88.7 ± 8.3% of baseline, *p* = 0.583 vs. control input, Fig. 2c). In contrast, bath perfusion of apocynin after LTD induction failed to reverse the expression of LTD, indicating that NOX2 is not required for LTD maintenance (apocynin perfusion: 68.8 ± 4.0% of baseline vs. control: 78.9 ± 3.4% of baseline, *p* = 0.078, Supplementary Fig. S1). Together, these data are suggestive of a specific role for the NOX2 isoform of NOX in the regulation of LTD induction.

Owing to the limited pharmacological selectivity and off-target effects of available NOX inhibitors, we next utilized short hairpin RNA (shRNA) to knockdown either NOX1 or 2 expression (Fig. 2d). Biolistic transfection of NOX1 or NOX2 shRNA into CA1 neurons of organotypic cultured hippocampal slices was performed. LTD was readily inducible in cells transfected with NOX1 shRNA (NOX1 shRNA: 54.9 ± 4.7% of baseline; untransfected cells: 61.7 ± 3.5% of baseline, *p* = 0.280, Fig. 2e). In contrast, LTD was abolished in neurons transfected with NOX2 shRNA (NOX2 shRNA: 87.3 ± 11.5% of baseline; untransfected cells: 50.7 ± 7.4% of baseline, *p* = 0.028, Fig. 2f). Neither NOX1 nor NOX2 shRNA influenced the basal state of glutamatergic synaptic transmission, as measured by AMPAR-mediated excitatory postsynaptic current (EPSC_AMPA_; Supplementary Fig. S2a and S2c) and NMDAR-mediated EPSC (EPSC_NMDA_; Supplementary Fig. S2b and S2d). Furthermore, transfection of a scrambled form of NOX2 shRNA had no effect on LTD (scrNOX2 shRNA: 54.9 ± 7.3% of baseline; untransfected cells: 49.3 ± 5.2% of baseline, *p* = 0.541, Supplementary Fig. S2e). Therefore, our data strongly suggest that ROS production through postsynaptic NOX2 is involved in LTD regulation.

### PKCζ and phosphorylation of p47phox is required for LTD

**Fig. 3:**
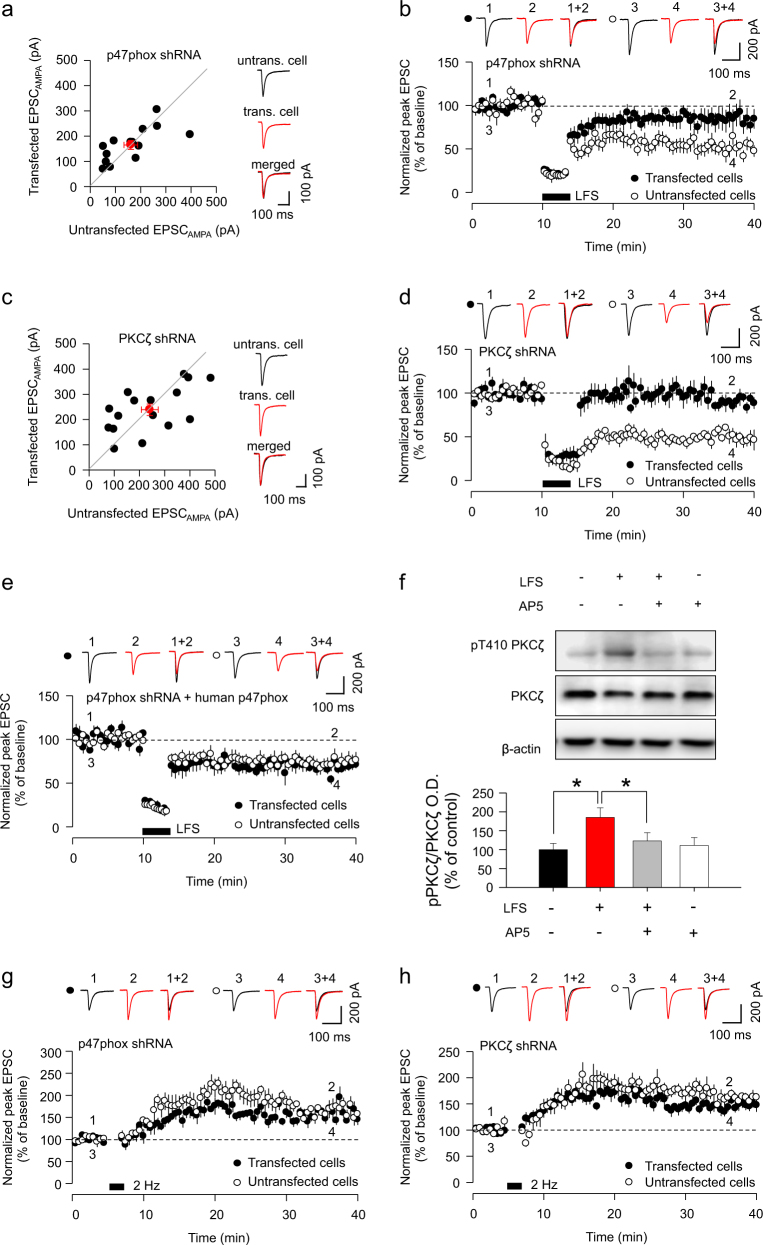
P47phox and PKCζ are necessary for LTD. **a** Synaptic AMPAR-mediated currents (EPSC_AMPA_) are unchanged between p47phox shRNA-transfected cells and neighbouring untransfected cells (*n* = 13 pairs). Individual data points represent individual pairs of neurons. Red circles represent mean ± SEM. **b** LTD is abolished in neurons transfected with p47phox shRNA (filled circle, *n* = 9), whilst LTD is induced in untransfected cells (open circle, n = 8). **c** Synaptic AMPAR-mediated currents (EPSC_AMPA_) are unchanged between PKCζ shRNA-transfected cells and neighbouring untransfected cells (*n* = 16 pairs). Individual data points represent individual pairs of neurons. Red circles represent mean ± SEM. **d** LTD is abolished in neurons transfected with PKCζ shRNA (filled circle, *n* = 7), whilst LTD is induced in untransfected cells (open circle, *n* = 6). **e** Co-transfection of human p47phox with p47phox shRNA rescues the expression of LTD (filled circles, *n* = 7) to levels similar to untransfected cells (open circles, *n* = 7). **f** Immunoblots showing LFS-induced enhanced PKCζ phosphorylation at threonine 410 residue (pT410PKCζ; *n* = 5) and AP5 inhibition of LFS-induced pT410PKCζ (*n* = 5). **g** LTP was present in p47phox shRNA-transfected cells (filled circle, *n* = 7) and untransfected cells (open circle, *n* = 5). **h** LTP was readily induced in both PKCζ shRNA-transfected cells (filled circle, *n* = 7) and untransfected cells (open circle, *n* = 7). Data represents mean ± SEM. Asterisk (*) indicates statistical significance (*p* < 0.05)

The function of NOX1/NOX2 is principally regulated through multi-site phosphorylation of the p47phox subunit of the complex by PKC isoforms, including the atypical PKCζ^27, 28^. Given the requirement for NOX2 in LTD, we hypothesized that phosphorylation of p47phox would also be required. To test this, we first utilized biolistic transfection of p47phox shRNA in CA1 neurons of organotypic hippocampal slices to knock down the expression of p47phox (Supplementary Fig. S3). Transfection of p47phox shRNA had no significant effect on basal EPSC_AMPA_ compared with untransfected neighbouring cells (untransfected: 145.0 ± 15.1 pA, transfected: 162.1 ± 15.1 pA, *p* = 0.594, Fig. 3a). Consistent with our hypothesis, p47phox shRNA transfection significantly impaired LTD expression when compared to untransfected cells (p47phox shRNA-transfected cells: 87.2 ± 11.5% of baseline; untransfected cells: 50.6 ± 7.1% of baseline, *p* = 0.0145, Fig. 3b).

Since phosphorylation of the p47phox subunit by the atypical PKCζ leads to superoxide generation^16, 27^, we therefore used PKCζ shRNA (Supplementary Fig. S4) to examine whether PKCζ is also required for LTD. We found that transfection of PKCζ shRNA had no significant effect on basal EPSC_AMPA_ (untransfected: 241.0 ± 33.2 pA, transfected 238.9 ± 22.6 pA, *p* = 0.957, Fig. 3c) but significantly impaired LTD (PKCζ shRNA: 90.6 ± 4.4% of baseline; untransfected cells: 50.4 ± 5.4% of baseline, *p* = 0.0001, Fig. 3d). Furthermore, the p47phox shRNA-mediated LTD deficit was rescued by co-expression of human p47phox (untransfected cells: 74.8 ± 8.9% of baseline; transfected cells: 69.2 ± 4.5% of baseline, *p* = 0.583; Fig. 3e).

To substantiate our hypothesis that PKCζ is an active component of LTD signalling, we tested whether LFS of hippocampal slices, which leads to NMDAR activation and LTD induction, regulates PKCζ activity. To do this, we utilized acutely prepared hippocampal slices prepared from 3-week-old rats. Since PKCζ activity depends on the phosphorylation of threonine 410 (pT410)^29, 30^, we analysed whether LFS phosphorylates PKCζ at pT410. We found that LFS of Schaffer collateral afferents significantly increased phosphorylation of PKCζ at pT410, indicative of PKCζ activation, and this was blocked by AP5 (50 μM), an NMDAR antagonist (Control: 100 ± 16%; LFS: 185 ± 25%, *p* < 0.05 compared to control; LFS+AP5: 123 ± 21%, *p* < 0.05 compared to LFS; AP5: 111 ± 21%, Fig. 3f).

Interestingly, long-term potentiation (LTP) was readily inducible in cells expressing p47phox shRNA (p47phox shRNA: 154.2 ± 6.5% of baseline; untransfected cells: 167.1 ± 14.9% of baseline, *p* *=* 0.416, Fig. 3g). LTP was also intact in cells expressing PKCζ shRNA (PKCζ shRNA: 147.9 ± 9.4% of baseline; untransfected cells: 160.1 ± 10.0% of baseline, *p* = 0.391, Fig. 3h), indicating a selective role for p47phox and PKCζ in the LTD form of synaptic plasticity. Together, these data indicate that PKCζ activation downstream of NMDAR activation is a fundamental component of LTD signalling, regulating the activity of NOX2 via p47phox.

### Phosphorylation of p47phox at serine 316 is required for LTD

**Fig. 4:**
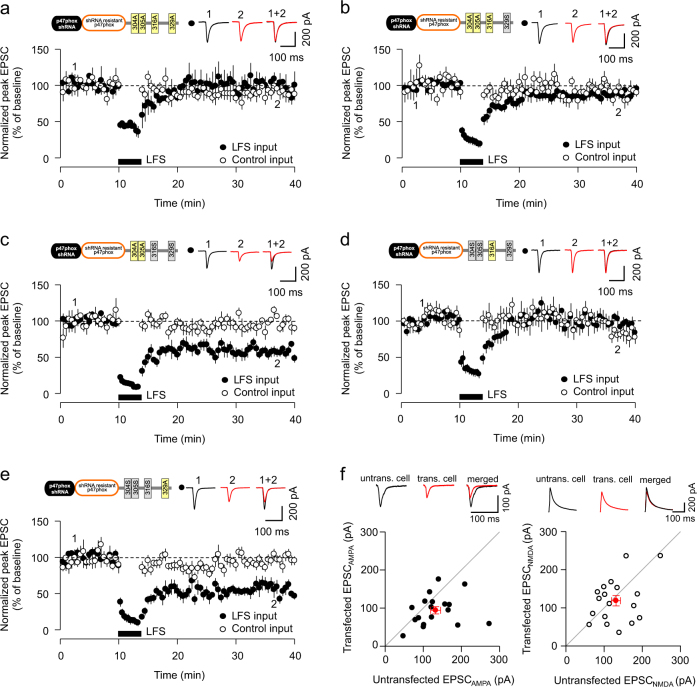
Phosphorylation of p47phox at S316 is required for LTD. **a** LTD is impaired in cells co-transfected with p47phox shRNA and p47phox shRNA-resistant form of the S/T304/305/316/329A phosphorylation mutant p47phox (*n* = 7). **b** LTD is impaired in cells co-transfected with p47phox shRNA and p47phox shRNA-resistant form of the S304/305/316A triple phosphorylation mutant p47phox (*n* = 7). **c** LTD is inducible in cells co-transfected with the S304/305A double mutant form of p47phox (*n* = 7). **d** LTD is impaired in cells co-transfected with the S316A mutant form of p47phox (*n* = 10). **e** LTD is inducible in cells co-transfected with the S329A mutant form of p47phox (*n* = 7). Data represents mean ± SEM. **f** Synaptic AMPAR-mediated currents (EPSC_AMPA_, filled circles), but not NMDAR-mediated currents (EPSC_NMDA_, empty circles), are reduced in cells transfected with p47phox S316D compared to neighbouring untransfected cells (*n* = 18 pairs). Individual data points represent individual pairs of neurons. Red circles represent mean ± SEM. Representative single traces are provided in inset

Since both p47phox shRNA and PKCζ shRNA transfection resulted in a loss of LTD, it was of interest to determine the specific molecular mechanism surrounding the regulation of p47phox by PKCζ. We generated shRNA-resistant constructs of rat p47phox with differing combinations of site-specific mutations at four residues phosphorylated by PKCζ^27^. Consistent with our working hypothesis, LTD was blocked in cells co-transfected with p47phox shRNA and an shRNA-resistant mutant form of p47phox with all four residues mutated to alanine, to prevent their phosphorylation (after LFS: 95.1 ± 6.8%, *p* = 0.725 vs. control input, Fig. 4a). In cells expressing a triple phosphorylation mutant (S/T304/305/316A—residues notated as per rat p47phox), LTD was also impaired (after LFS: 88.0 ± 5.5%, *p* = 0.087 vs. control input, Fig. 4b). In comparison, LTD was readily inducible in cells transfected with a double S/T304/305A mutant (after LFS: 57.2 ± 4.1%, *p* = 0.000117 vs. control input, Fig. 4c). Notably, we found that LTD was also impaired in cells expressing the S316A mutant form of p47phox (after LFS: 98 ± 22.8%, *p* = 0.73 vs. control input, Fig. 4d) but the expression of S329A mutant form of p47phox had no effect on LTD induction (after LFS: 63.1 ± 7.0%, *p* = 0.004 vs. control input, Fig. 4e). Finally, we tested whether phosphorylation of p47phox at S316 is sufficient to induce synapse weakening, through paired recordings from neurons transfected with a S316 pseudo-phosphorylated form of p47phox (S316D) and neighbouring untransfected neurons. Our data showed significantly reduced AMPAR-mediated currents in S316D-transfected neurons (untransfected: 132.4 ± 12.9 pA vs. S316D transfected: 94.1 ± 9.4 pA, *p* = 0.023, Fig. 4f), indicating that phosphorylation at this residue is sufficient to reduce AMPAR-mediated synaptic transmission. This effect appears specific to AMPARs, as NMDAR-mediated currents were unchanged between the two cell types (untransfected: 131.1 ± 11.7 pA vs. S316D transfected: 118.7 ± 13.7 pA, *p* = 0.495, Fig. 4f). Taken together, these data suggest that phosphorylation at S316 of p47phox, likely by PKCζ, is a key regulator of a signalling cascade that governs LTD induction and synapse weakening.

## Discussion

The notion that ROS production is a key element of synaptic plasticity has been well established^11–13, 20, 31^. However, the precise source of ROS and mechanisms by which it is regulated during synaptic plasticity have not been fully elucidated. Identification of these specific signalling pathways is necessary for our full understanding of its physiological and pathophysiological implications in synaptic function.

In the present study, we have shown that postsynaptic NOX2-mediated superoxide production, via PKCζ-mediated phosphorylation of p47phox at the serine 316 residue (pS316 p47phox), is pivotal for LTD expression and weakening of AMPAR-mediated synaptic transmission. Importantly, few studies have directly addressed the source of ROS in the context of synaptic plasticity. Using our selective knockdown approach, in which we have specifically silenced or inhibited postsynaptic ROS production, we have now shown that postsynaptic NOX2 is a necessary hub for ROS production associated with LTD. Several research groups have shown a similar requirement for ROS during LTD or synapse weakening^9, 10, 12, 13^ whilst evidence from other groups suggests that ROS can regulate LTP^12, 31, 32^. Quite how or why ROS can regulate both forms of synaptic plasticity is unclear, but it is possible that different ROS production sources and mechanisms may underpin different forms of synaptic plasticity.

NMDAR activation leads to PKCζ-mediated phosphorylation of the p47phox subunit, which is a critical activator signal for NOX2 mediated ROS production^15, 16^. In the present study, we show that low-frequency electrical stimulation of hippocampal slices, which induces an NMDAR-dependent form of LTD, also leads to the activation of PKCζ. This effect was blocked by D-AP5, indicative of synaptic NMDAR-dependent activation of PKCζ that is associated with LTD induction. Importantly, through postsynaptic knockdown of PKCζ expression, we reveal an LTD-specific requirement for PKCζ with no observable contribution to LTP expression, consistent with other synapse weakening signals shown in previous studies^5, 6, 33–36^. Finally, our data show that constitutive phosphorylation of the PKCζ substrate, p47phox, at S316 is sufficient to induce weakening of AMPAR-mediated synaptic transmission, even in the absence of upstream activator signals. This single phosphorylation event, which induces NOX2 activation and ROS production, is therefore both necessary and sufficient for synapse weakening. It is not clear how postsynaptic ROS production is itself involved in the mechanisms of LTD signalling. One possibility is that postsynaptic ROS activates Bax protein to stimulate cytochrome *c* release from mitochondria^37^. Cytochrome *c* release induces caspase-3 activation^38^, which in turn can affect synapse weakening signal cascades involving Akt-1 and GSK-3β^4, 39^. This possibility is supported by the observation that Bax is itself required for LTD signalling^33^.

A growing list of molecules, including caspases, GSK-3β and tau, are now known to be involved in AMPAR endocytosis and LTD^5, 6, 33, 34^. Collectively, these molecules form what we have termed the synapse weakening pathway, encompassing molecules associated with apoptosis and synapse elimination in both physiological and pathophysiological circumstances^4, 36, 40^. It has been postulated that the balance between synapse weakening (caspase-3, GSK-3β and tau) and strengthening pathways (phosphoinositide-3 kinase and Akt-1) is critical to determine the direction of LTP and LTD and long-term fate of synapses^4–6, 35^. Indeed, models of neurodegenerative pathologies such as AD exhibit AMPAR endocytosis and facilitated LTD induction, concomitant with the inhibition of LTP in the hippocampus^2, 3, 39, 41, 42^. Aberrant activation of synapse weakening signals is therefore believed to be a central underlying molecular mechanism in the pathology and cognitive decline of numerous neurodegenerative diseases^4, 40, 43, 44^. Our results suggest that ROS, via a specific production mechanism, now form a part of this critical synapse weakening signalling cascade. Addressing whether aberrant LTD-like and/or AMPAR-mediated synapse weakening can be seen in human forms of neurodegenerative disease remains a key challenge to translating these findings into viable therapeutic targets.

## Materials and methods

### Animals

All procedures involving animals were carried out in accordance with the UK Animals (Scientific Procedures) Act, 1986. Male Wistar rats (Charles River, UK) were used to prepare organotypic (6–8 day-old rats) and acute hippocampal slices (2–4-week-old rats). Older rats were housed four or five per cage and allowed access to water and food ad libitum. The cages were maintained at a constant temperature (23 ± 1 °C) and relative humidity (60 ± 10%) under a 12-h light/dark cycle (lights on from 07:30 to 19:30).

### Acute hippocampal slices

Rats were killed by cervical dislocation and decapitation. Following this, the brain was rapidly removed and placed into ice-cold artificial cerebrospinal fluid (aCSF; continuously bubbled with 95% O_2_/5% CO_2_) containing 124 mM NaCl, 3 mM KCl, 26 mM NaHCO_3_, 1.25 mM NaH_2_PO_4_, 2 mM CaCl_2_, 1 mM MgSO_4_, and 10 mM d-glucose. Hippocampi were extracted and transverse hippocampal slices (400 µm thickness) were cut using a McIlwain tissue chopper. Following manual separation, the slices were then submerged in aCSF for a minimum of 1 h before experiments commenced.

Acute slices were placed in a recording chamber and perfused with warmed (28–29 °C) and carbogenated aCSF at 2 ml/min. Two independent stimulating electrodes were placed separately in the Schaffer collateral–CA1 input (test pathway) and subiculum–CA1 input (control pathway). For whole-cell patch experiments, 20 μM picrotoxin was included in the aCSF and CA1 neurons were blind-patched using a 4–6 MΩ borosilicate glass pipette containing 130 mM CsMeSO_4_, 8 mM NaCl, 4 mM Mg-ATP, 0.3 mM Na-GTP, 0.5 mM EGTA, 10 mM HEPES, 6 mM QX-314, pH 7.2–7.3 and 280–290 mOsm/kg. For field excitatory postsynaptic potential (fEPSP) recordings, a glass pipette containing 3 M NaCl was placed in the *stratum radiatum* region of the CA1. LTD experiments during whole-cell patch were carried out as for cultured hippocampal slices (see below), except a 20-min baseline was used. For LTD of fEPSPs, a 30-min stable baseline at 70% of maximum stimulation intensity was followed by LFS, consisting of 900 pulses at 1 Hz, of the test pathway. This was followed by 60 min of post-conditioning recording. In some cases, slices were removed immediately after LFS for western blotting processing.

### Hippocampal slice culture and whole-cell patch recording

Hippocampal slice cultures were prepared from 6–8-day-old male Wistar rats. Hippocampal slices (350 μm) were cut using a McIlwain tissue chopper and cultured on semipermeable membrane inserts (Millipore Corporation, Bedford, MA, USA) in a six-well plate containing culture medium (78.8% minimum essential medium, 20% heat-inactivated horse serum, 30 mM HEPES, 26 mM d-glucose, 5.8 mM NaHCO_3_, 2 mM CaCl_2_, 2 mM MgSO_4_, 70 μM ascorbic acid, 1 μg/ml insulin, pH adjusted to 7.3 and 320–330 mOsm). Slices were cultured for 6–8 days in vitro (DIV) with a change of medium every 2 days, without antibiotics. Neurons were transfected using a biolistic gene gun (Helios Gene-gun system, Bio Rad, USA) at DIV3–4 (100 μg DNA; 90% of the construct to test; 10% pEGFP-C1). Electrophysiological recordings were performed 3–4 days after transfection.

For whole-cell patch recordings, cultured slices were perfused with a warmed (28–29 °C) recording solution (119 mM NaCl, 2.5 mM KCl, 26 mM NaHCO_3_, 1 mM NaH_2_PO_4_, 4 mM MgCl_2_, 11 mM d-glucose, 4 mM CaCl_2_, 10 μM 2-chloroadenosine and 20 μM picrotoxin). The recording solution was continuously bubbled with 95% O_2_/5% CO_2_ at source. The usual flow rate was 2 ml/min. In most recordings, two independent stimulating electrodes were placed separately in the Schaffer collateral–CA1 input and subiculum–CA1 input. Recordings were made from pyramidal neurons in the CA1 region, using glass pipettes containing CsMeSO_4_ internal solution (as above) and neurons voltage clamped at −70 mV unless otherwise stated. To induce LTD, a 10-min baseline was followed by 1 Hz stimulation (200 stimuli) with recorded neurons voltage clamped at −40 mV. To induce LTP, a 5-min baseline was followed by a 2 Hz stimulation (200 stimuli) with recorded neurons voltage clamped at 0 mV. For quantification and comparisons between groups/inputs, the peak EPSC amplitude of the test input (relative to baseline) was averaged 15–20 min (cultured slice) or 20–25 min (acute slice) after conditioning was applied. EPSC_AMPA_ was measured as the peak EPSC amplitude at a holding potential of −70 mV. EPSC_NMDA_ was measured as the peak EPSC amplitude 90–100 ms after stimulus, at a holding potential of +40 mV.

### Drugs and antibodies

The following drugs were dissolved in internal recording solution (for postsynaptic infusion) or aCSF (for bath perfusion) at concentrations based on previous studies: AEBSF (20 μM^45^; Sigma-Aldrich, MO, USA), apocynin (100 μM^46^; Abcam, Cambridge, UK), SOD (300 units/ml^47^; Sigma-Aldrich, MO, USA), catalase (300 units/ml^48^; Sigma-Aldrich, MO, USA), ML-171 (3 μM^49^; Tocris, Oxford, UK) and AP5 (Hello Bio, Bristol, UK). Primary antibodies used for western blotting were pT410 PKCζ (Cell Signaling, MA, USA), total PKCζ (Santa Cruz, Texas, USA) and total β-actin (Abcam, Cambridge, UK).

### Expression/shRNA plasmids and single-cell PCR

Constructs for shRNA knockdown of target transcripts were generated using the Block-iT™ pENTR/U6 system, as per the manufacturer’s instructions (Life Technologies, UK). The target sequences for rat NOX1 and NOX2 were GCAACTGTTCATACTCTTTCC and GGTCTTACTTTGAAGTGTTCT, respectively. NOX2 scrambled shRNA sequence was GGTAGTTACTCGTTAGTTTCT. PKCζ shRNA target sequence was GGCCATGAGCATCTCTGTTGT and for p47phox it was GCTCCTACCCTGCTTTAATGT. P47phox-rescue and mutation experiments were performed with co-transfection of the p47phox shRNA construct and a pCMV-SPORT6-p47phox cDNA clone (Source Bioscience, UK). Site-directed mutagenesis to generate S/T304/305A, S/T304/305/316A, S316A and S329A constructs was performed on the pCMV-SPORT6-p47phox construct using QuikChange™ technology, as per the manufacturer’s instructions (Agilent Technologies, USA). All generated constructs were sequence verified via Sanger sequencing (Source Bioscience, UK).

Knockdown efficiencies of the generated shRNAs were verified by neuronal single-cell PCR. Briefly, transfected and non-transfected control cells were excised from cultured slices using low-resistance electrodes and then snap frozen. Cells were then subjected to OneStep™ RT-PCR (Qiagen, The Netherlands), as per the manufacturer’s instructions, using specific exon-spanning primers: NOX1: sense 5′-AGAGGCTCCAGACCTCCATTT-3′, anti-sense, 5′-CGTGTGGTTGCAAAATGAGCA-3′; NOX2: sense 5′-AGCACTTCACACGGCCATTC-3′, anti-sense 5′-AGAGGTCAGGGTGAAAGGGT-3′; PKCζ: sense 5′-GGACCTCTGTGAGGAAGTGC-3′, anti-sense, 5′-GGATGCTTGGGAAAACGTGG-3′; PKMζ: sense 5′-CCTTCTATTAGATGCCTGCTCTCC-3′, anti-sense 5′-TGAAGGAAGGTCTACACCATCGTTC-3′^50^; p47phox: sense 5′-CACCTTCATTCGCCACATCG-3′, anti-sense 5′-ATGTCCCTTTCCTGACCAC-3′; Cyclophilin: sense 5′-CCAAGACTGAGTGGCTGGAT-3′, anti-sense 5′- TCCTGCTAGACTTGAAGGGGAA-3′. Amplified PCR products were resolved on a 1% agarose/Midori Green gel and analysed under ultraviolet light.

### Statistical analyses

Sample sizes, indicated by *n*, are indicated in the figure legends and represent the number of biological replicates. For experiments in acute slices, this reflects the number of individual animals from which the data were obtained. For cultured slices, this reflects the number of individual slices. Samples sizes for electrophysiology experiments were determined through empirical evidence obtained within our laboratory and are consistent with those found in existing literature. Data are expressed as mean ± standard error of the mean (SEM) and analysed using the SigmaPlot software (Systat Software, Chicago, USA). Significance was set at *p* < 0.05 and unpaired *t* tests were used to determine the statistical significance of effects vs. control inputs or untransfected cells, where appropriate, and paired *t* tests were used to compare to baseline values, when necessary.

## Electronic supplementary material

Supplemental Material File #1

## Electronic supplementary material

**Supplementary Information** accompanies the paper at (10.1038/s41421-018-0046-x).
