# Supplementary material for: Postsynaptic p47phox regulates long-term depression in the hippocampus
Source: Cell Discov. 2018 Aug 28;4:44. doi: 10.1038/s41421-018-0046-x (PMC6110736; doi:10.1038/s41421-018-0046-x)
Supplement: Supplementary file 1 — Supplemental Material File #1 [file 41421_2018_46_MOESM1_ESM.pdf]

Supplementary Figure 1

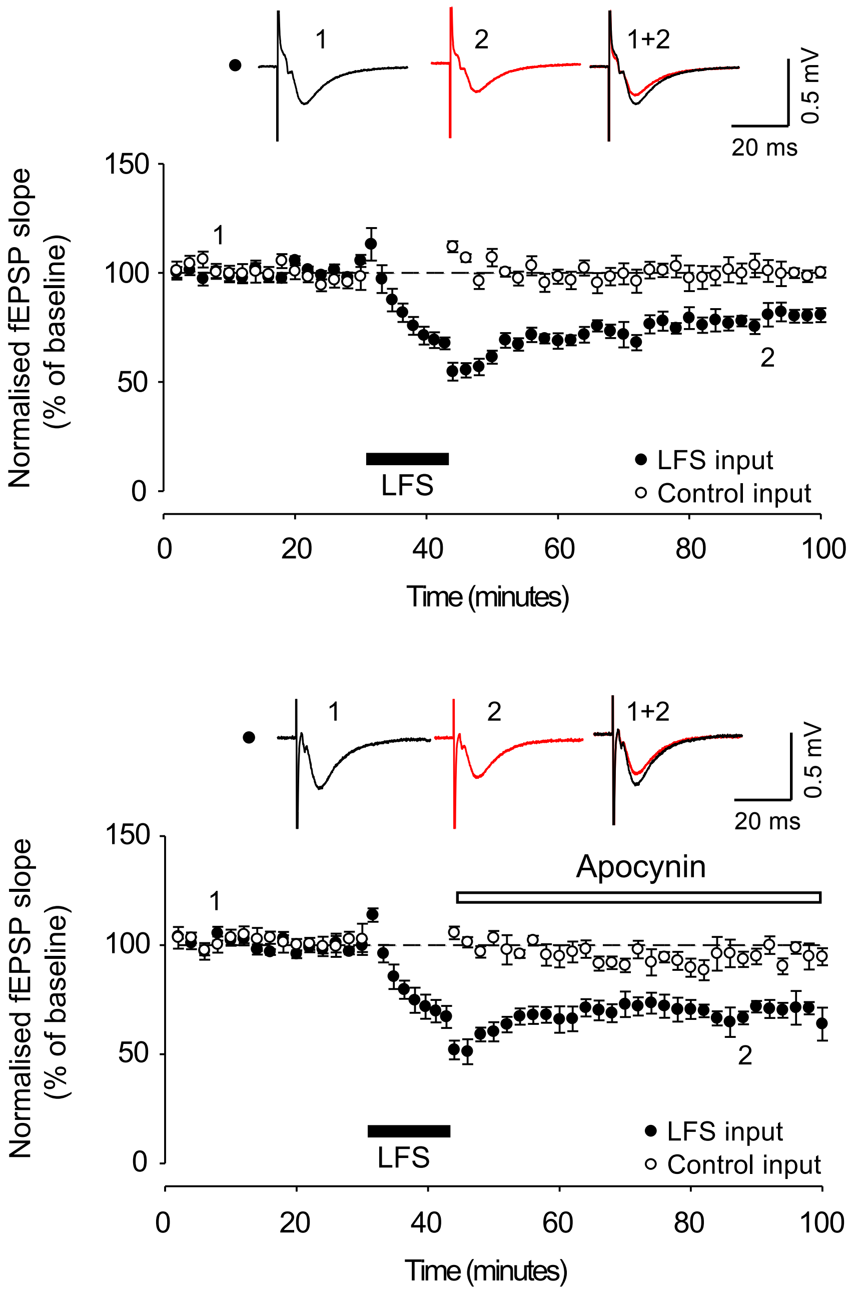

Supplementary Figure 2

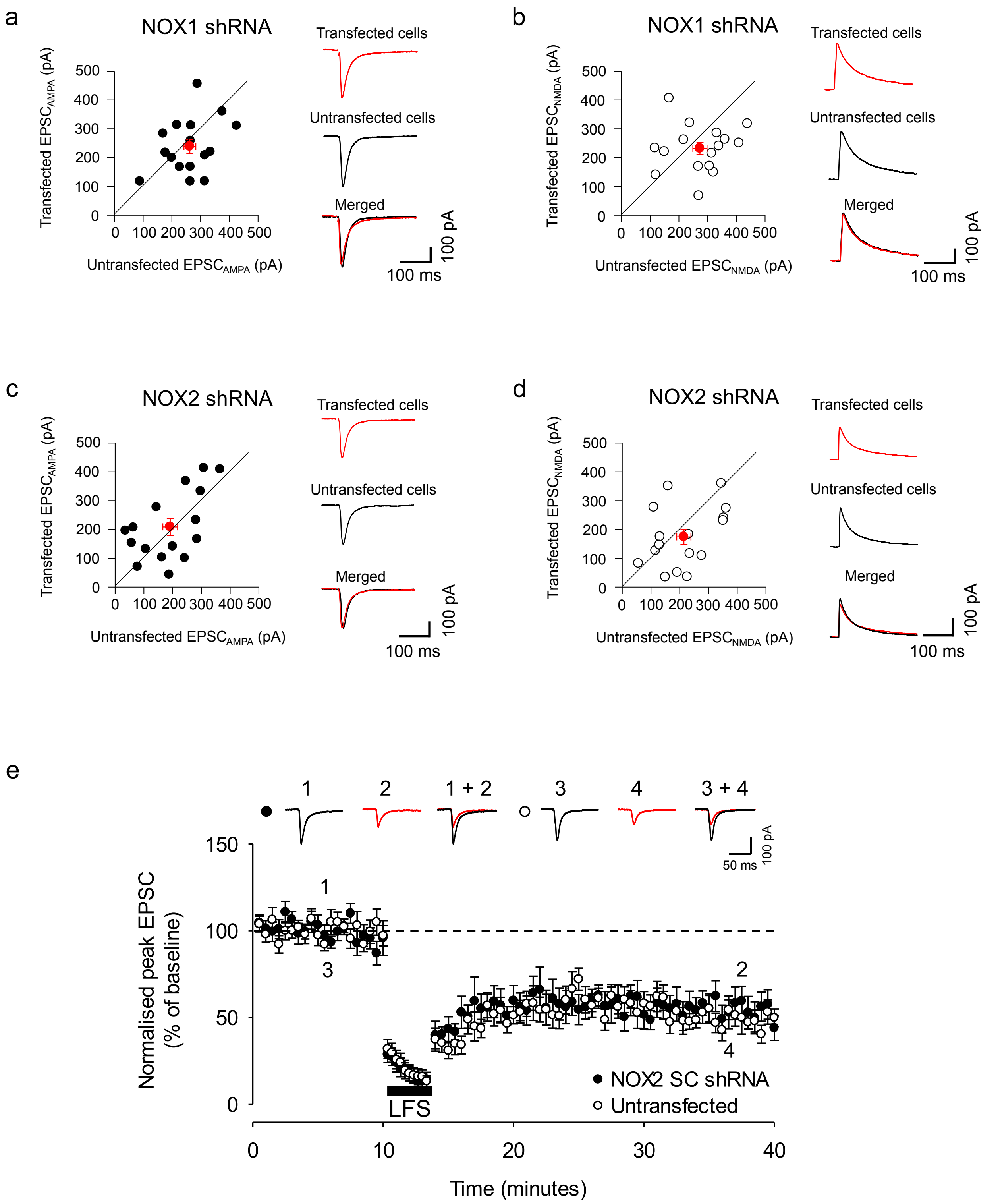

Supplementary Figure 3

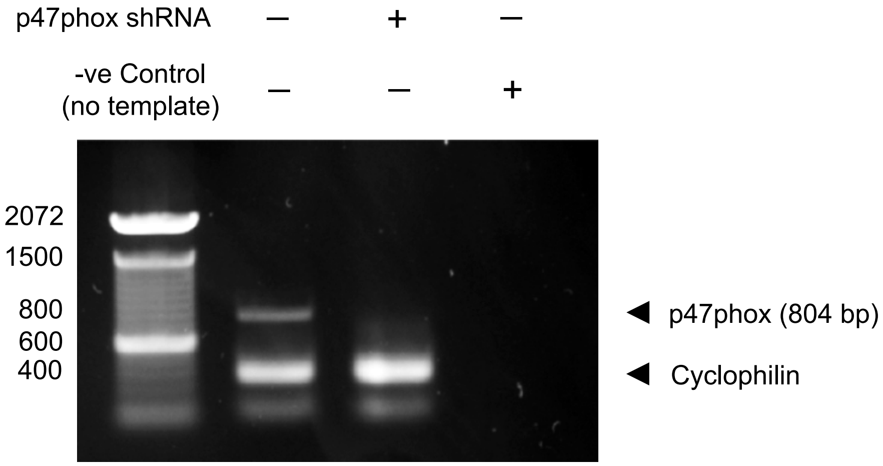

Supplementary Figure 4

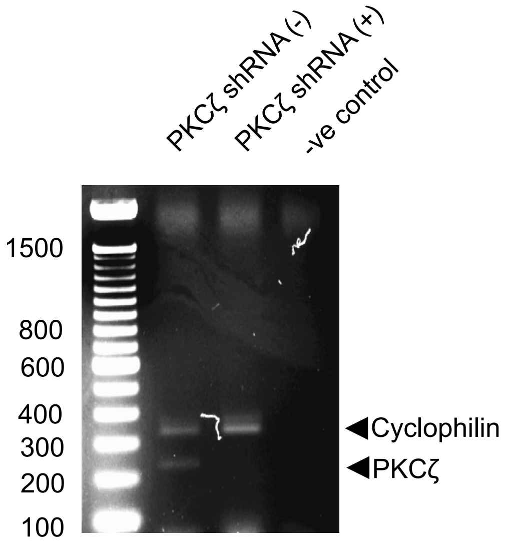

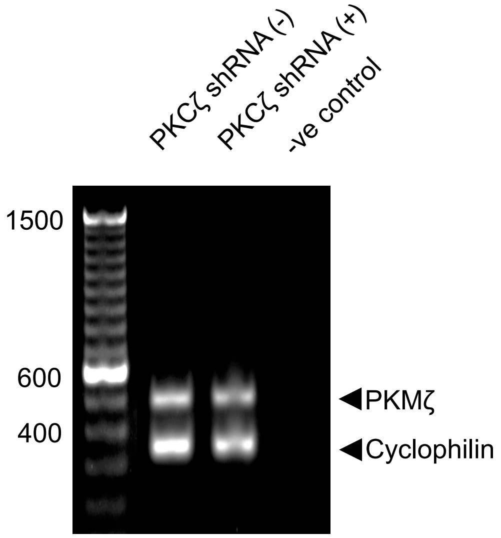

Supplementary Figure Legends

Supplementary Fig. 1. Bath perfusion of apocynin after LTD induction has no effect on LTD expression (control: n = 7; apocynin: n = 6).

Supplementary Fig. 2. Transfection of NOX1 shRNA has no effect on $EPSC_{AMPA}$ (**a**) and $EPSC_{NMDA}$ (**b**) compared to neighbouring untransfected neurons (n = 16 pairs). Transfection of NOX2 shRNA has no effect on $EPSC_{AMPA}$ (**c**) and $EPSC_{NMDA}$ (**d**) compared to neighbouring untransfected neurons (n = 16 pairs). Individual data points represent individual pairs of neurons. Red circles represent mean ± SEM. Representative single traces are provided inset. (**e**) LTD is induced in neurons transfected with scrambled NOX2 shRNA (n = 10) and untransfected cells (n = 10).

Supplementary Fig. 3. Single cell PCR assay demonstrating efficient knockdown p47Phox shRNA.

Supplementary Fig. 4. Single cell PCR assays demonstrate efficient and selective knockdown of PKCζ (left) with PKCζ shRNA, and this has no effect on PKMζ (right).
